# Avançando no Diagnóstico e Tratamento da Insuficiência Cardíaca com Fração de Ejeção Preservada: Um Alerta sobre a Necessidade do Estudo Hemodinâmico com Exercício

**DOI:** 10.36660/abc.20230845

**Published:** 2024-07-31

**Authors:** Renato de Aguiar Hortegal, Fausto Feres

**Affiliations:** 1 Instituto Dante Pazzanese de Cardiologia São Paulo SP Brasil Instituto Dante Pazzanese de Cardiologia, São Paulo, SP – Brasil

**Keywords:** Insuficiência Cardíaca, Testes de Função Cardíaca, Ecocardiografia

## Introdução

A insuficiência cardíaca (IC) é uma condição médica progressiva que afeta aproximadamente 1-3% dos adultos, com aumento da expressivo da prevalência em populações com faixa etária mais elevada.^1^ Mais da metade dos pacientes com IC apresenta fração de ejeção do ventrículo esquerdo (FEVE) ≥ 50%, conhecida como insuficiência cardíaca com fração de ejeção preservada (ICFEP). Embora o número global de casos de IC permaneça estável ou mesmo em declínio, a incidência de ICFEP continua aumentando.^2^

Pacientes com ICFEP diferem significativamente daqueles com fração de ejeção reduzida quanto à fisiopatologia, avaliação diagnóstica e ao tratamento. A heterogeneidade fisiopatológica idiossincrática e a disfunção de múltiplos órgãos, além da apresentação clínica diversificada, requerem abordagens personalizadas para esta população.

Considerando tais aspectos, uma unidade dedicada a atender pacientes com ICFEP foi fundada em Outubro de 2020 no Instituto Dante Pazanese de Cardiologia, um centro de saúde pública terciário e quartenário localizado em São Paulo, Brasil. Até o momento, esta unidade realizou mais de 5.000 consultas ambulatoriais, predominantemente para pacientes que apresentam dispneia induzida pelo exercício.

Nesse cenário, nos deparamos com um desafio significativo da Cardiologia moderna^3^: desenvolver metodologias para uma avaliação clínica eficaz e prática para o diagnóstico de ICFEP.

Os critérios diagnósticos atuais apresentam considerável heterogeneidade e falta de intercambialidade.^4,5^ De fato, a limitação em estabelecer um diagnóstico preciso pode prejudicar diversas intervenções terapêuticas já testadas em estudos multicêntricos. Esses aspectos do campo de pesquisa têm implicações diretas sobre a forma como os médicos podem lidar com as queixas de dispneia encontradas na prática diária em pacientes com suspeita de ICFEP.

### Uso de escores para ICFEP

Pacientes com ICFEP podem divergir da apresentação clássica de IC, já que aproximadamente 50% deles apresentam fenótipo caracterizado por hipertensão atrial esquerda induzida por exercício, manifestando sintomas exclusivamente durante o esforço, sem sinais atuais ou prévios de sobrecarga hídrica ao exame clínico ou internação prévia.^6,7^

Para tornar a situação ainda mais complexa, 20-35% dos pacientes com ICFEP apresentam níveis normais de peptídeos natriuréticos,^1,8^ 29% não apresentam anormalidades estruturais à ecocardiografia^4^ e a presença de disfunção diastólica na ecocardiografia não é específica nem suficiente para fechar um diagnóstico conclusivo.^1,6^

**Figura 1:**
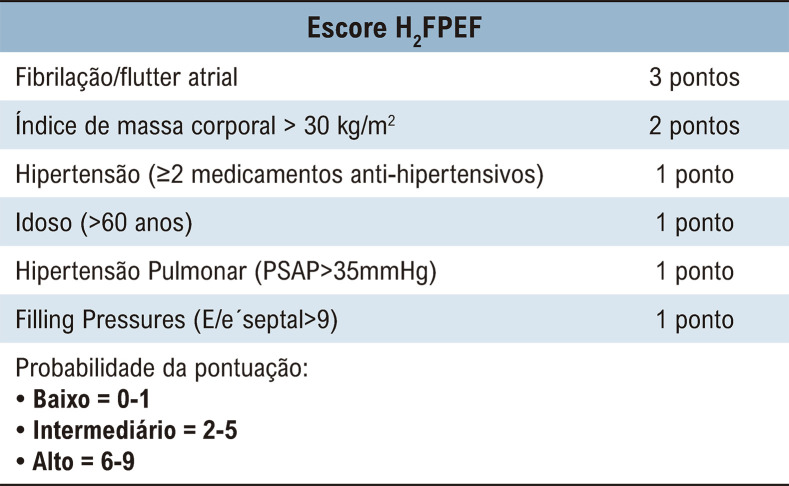
– Escore H2FPEF para estimar a probabilidade de ICFEP

**Figura 2:**
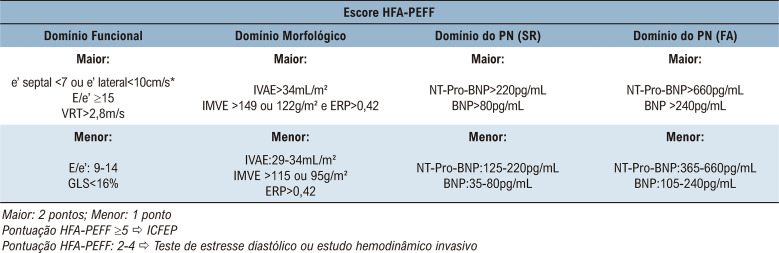
– Pontuação de HFA-PEFF para avaliação do diagnóstico de ICFEP. HFA-PEFF: Associação de Insuficiência Cardíaca-PEFF; PN: peptídeo natriurético; e’: velocidade diastólica precoce do anel mitral; E: velocidade de fluxo transmitral precoce; VRT: velocidade de regurgitação tricúspide; TLG: tensão longitudinal global do ventrículo esquerdo; IVAE: índice de volume atrial esquerdo; IMVE: índice de massa ventricular esquerda; ERP: espessura relativa da parede; VE: ventrículo esquerdo; RS: ritmo sinusal; NT-proBNP: peptídeo natriurético pró-tipo B N-terminal; BNP: peptídeo natriurético tipo B; FA: fibrilação atrial. *Os valores devem ser ajustados para e’<5cm/s e e’ lateral<7cm/s se o paciente tiver idade>75 anos.

Neste contexto, dois escores desenvolvidos recentemente foram introduzidos com o objetivo de diagnosticar ICFEP.^9,10^ A pontuação H_2_FPEF é um nomograma validado por hemodinâmica invasiva que estima a probabilidade de ICFEP por meio da avaliação de características clínicas e variáveis de Doppler, com uma probabilidade pré-teste assumida de 64% (Figura 1). Por outro lado, o HFA-PEFF é um escore (Figura 2) proposto com validação *post-hoc* em duas grandes coortes.^11^

A incorporação desses escores em documentos oficiais de cardiologia é de grande importância, pois contribui para a padronização e rastreabilidade das avaliações diagnósticas. No entanto, é importante frisar que existem dados limitados sobre a validação externa e concordância destas pontuações.

**Figura 3:**
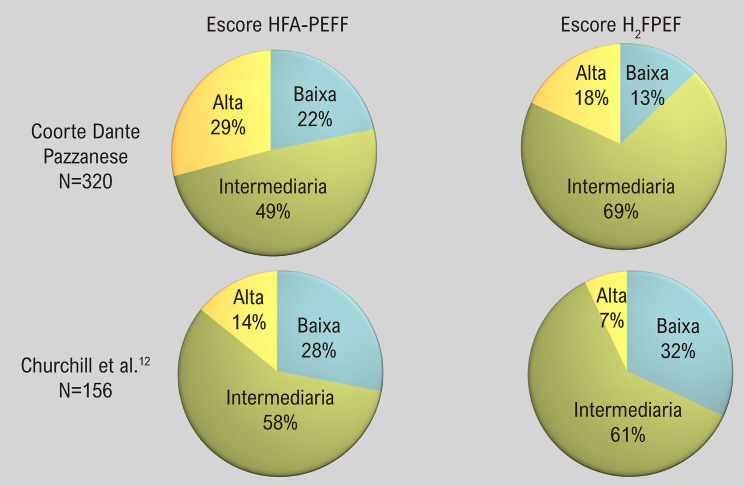
– Distribuição da probabilidade de ICFEP entre os pacientes de acordo com as pontuações de HFA-PEFF e H*2*FPEF.

Dados preliminares de nossa unidade, derivados de uma amostra de conveniência de 320 indivíduos com dispneia grau NYHA ≥2 e suspeita de ICFEP, produziram informações valiosas. A maioria dos pacientes avaliados apresentou pontuações que denotam probabilidade intermediária de ICFEP. Em particular, o escore H_2_FPEF exibiu uma proporção significativamente maior de probabilidade intermediária em comparação com o escore HFA-PEFF (69% vs. 49%, respectivamente) (Figura 3).

Outros autores também destacaram limitações destes escores: Churchill et al.^12^ relataram uma coorte de 156 indivíduos com dispneia crônica e FEVE≥ 50% submetidos ao teste cardiopulmonar invasivo (iCPET), em que identificaram uma pontuação HFA-PEFF de baixa probabilidade em 28%, intermediário em 58%, e alta em 14%; O H_2_FPEF foi baixo em 32%, intermediário em 61% e alto em 7% (Figura 3). Além disso, os autores relataram uma taxa de falsos negativos de 25% e 28% para baixa probabilidade em HFA-PEFF e H_2_FEPEF, respectivamente.

Tais achados têm duas implicações dignas de nota: 1) mais estudos são necessários para avaliar como melhorar o desempenho geral dos escores, especialmente para indivíduos com pontuações baixas e intermediárias e 2) a maioria dos pacientes com suspeita de ICFEP provavelmente necessitará de testes de exercício ou métodos alternativos de estresse hemodinâmico, como desafios de pré-carga e pós-carga.^13,14^ Diretrizes recentes recomendam Ecocardiografia com Teste de Estresse Diastólico e/ou Cateterismo Cardíaco Direito com Exercício para tais casos.^1,10^

### Limitações dos métodos não invasivos para diagnosticar ICFEP

O teste de estresse diastólico com ecocardiografia é o método não invasivo de escolha para investigar a suspeita de ICFEP através da provocação com exercício.^15,16^

No entanto, este método apresenta limitações significativas: a principal variável ecocardiográfica, a relação E/e’, pode apresentar problemas de medição durante o pico do exercício em até 20% dos casos. Além disso, a relação E/e’ medida durante o exercício pode gerar resultados falso-positivos em até 29% dos casos, além de apresentar acurácia sub-ótima.^17^

Apesar da Diretriz Europeia^10^ recomendar recalcular os escores, adicionando 2 pontos (quando E/e’ durante o exercício >14 + velocidade tricúspide durante o exercício < 3,4 m/s) ou 3 pontos (quando E/e’ durante o exercício >14 + velocidade tricúspide durante o exercício > 3,4 m/s) à pontuação do HFA-PEFF, esta abordagem carece de apoio baseado em evidências.

Embora essas limitações possam dificultar o uso da ecocardiografia de estresse diastólico na definição do diagnóstico de ICFEP, ela continua sendo um componente significativo dentro da abordagem multimodal mais ampla para pacientes com suspeita de ICFEP.

Análise das trocas gasosas por meio do teste de exercício cardiopulmonar (TECP) representa o padrão ouro para avaliação não invasiva da capacidade funcional. Este método explora a interação entre a mecânica pulmonar e as interações cardiopulmonares no contexto da fraqueza muscular.^18^ Além disso, dados recentes sugerem que as anormalidades na cinética do oxigênio podem ter estreita relação com alterações da na mecânica miocárdica.^15,19^

Embora o uso isolado do TECP não invasivo possa não ser suficiente para distinguir a ICFEP da dispneia não cardíaca em certos casos, o TECP desempenha um papel valioso como ferramenta diagnóstica para excluir doença pulmonar.^18^

### Cateterismo cardíaco direito como ferramenta diagnóstica para ICFEP

Dadas as limitações das modalidades diagnósticas não invasivas, um fator essencial capaz de oferecer informações críticas é a definição hemodinâmica única da síndrome da ICFEP. Esta definição estabelece a ICFEP como a incapacidade do sistema cardiovascular de manter um débito cardíaco adequado diante pressões de enchimento normais em repouso ou durante o exercício.

O cateterismo cardíaco direito (CCD) é o padrão-ouro para o diagnóstico de ICFEP, devido à sua capacidade de medir a pressão de oclusão da artéria pulmonar POAP.^20-22^ O procedimento apresenta baixo risco de complicações, com taxas relatadas inferiores a 1%. Além disso, demonstra excelente desempenho diagnóstico.^22^

Em condições de repouso, a POAP ≥ 15mmHg obtida pelo cateter Swan Ganz indica hipertensão atrial esquerda. A POAP em última análise, reflete a transmissão retrógrada da pressão média do átrio esquerdo para o capilar pulmonar.^23^ Quando elevada, mesmo que transitoriamente, pode causar congestão pulmonar.

Contudo, a normalidade da POAP medida em repouso não exclui o diagnóstico de ICFEP. Na realidade, muitos pacientes necessitarão de esforço físico para identificar anormalidades hemodinâmicas. Após as medições em repouso, o paciente fica em decúbito dorsal e inicia o exercício com o cicloergômetro, realizando medidas de POAP em intervalos de aproximadamente 2-3 minutos. Um valor de POAP ≥ 25mmHg durante o exercício define o diagnóstico de ICFEP.^22^

Uma definição alternativa considera a variação durante o exercício do aumento da POAP (slope da POAP) em relação ao aumento do débito cardíaco (slope do DC). Uma razão entre os slopes >2 (ou seja, slope POAP/slope DC>2) é indicativa de ICFEP.^24^

Vale mencionar que o CCD com exercício requer uma estrutura complexa e exige conhecimentos especializados para aquisição e interpretação de dados. Além disso, existem variações notáveis nos protocolos (ortostático vs. decúbito dorsal) e métodos de padronização para medir a POAP, como o ponto de referência (final da expiração da onda A média vs. média durante o ciclo respiratório).^22^

Em caso de limitações à execução do CCD com exercício, uma modalidade alternativa de estresse, como um desafio de pré-carga (prova de volume) pode ser valioso. Uma POAP ≥ 18mmHg induzida por elevação passiva da perna ou administração intravenosa de solução salina 7 mL/Kg é diagnóstica de ICFEP.^20,22^

### Cateterismo cardíaco direito para avaliação da extração periférica de oxigênio prejudicada: Um fator que contribui para a intolerância ao exercício na ICFEP

Na ICFEP, a capacidade dos músculos de extrair oxigênio da corrente sanguínea e utilizá-lo para processos metabólicos fica comprometida. Essa extração periférica prejudicada pode levar ao fornecimento inadequado de oxigênio aos músculos em exercício, resultando em fadiga precoce, redução da capacidade de exercício e dispneia.^25^

A avaliação da saturação de oxigênio na artéria pulmonar (Sa_o2_) e da saturação venosa mista de oxigênio (Sv_o2_) oferece informações sobre a extração de oxigênio em pacientes com ICFEP. Redução de Sa_o2_ e aumento de Sv_o2_, indicando utilização prejudicada de oxigênio durante o exercício. Essas medições podem ajudar a identificar pacientes com ICFEP que apresentam extração de oxigênio prejudicada, apesar dos níveis sistêmicos de oxigênio preservados.

A integração dos parâmetros hemodinâmicos obtidos pelo CCD permite avaliar diversas condições fisiológicas durante o exercício. Esses parâmetros incluem débito cardíaco, volume sistólico, resistência vascular pulmonar (RVP) e sistêmica, pressão arterial pulmonar (PAP) e extração periférica de oxigênio. A equação de Fick pode ser usada para avaliar cada componente capaz de impactar a capacidade de exercício:^25^


$$\begin{array}{cc} & {VO}_{2}=CO\times \left({CaO}_{2}-{CVO}_{2}\right)ou\\ & {VO}_{2}=SV\times HR\times 1,34\times Hb\times \left({SaO}_{2}-{SVO}_{2}\right) \end{array}$$

Onde VO_2_ representa o consumo de oxigênio, CO representa o débito cardíaco, Cao_2_ representa o conteúdo de oxigênio arterial, Cvo_2_ representa o conteúdo de oxigênio venoso, SV representa o volume sistólico, HR representa a frequência cardíaca, Hb representa a hemoglobina, Sao_2_ representa a saturação arterial de oxigênio e Svo_2_ representa a saturação venosa de oxigênio.

### Cateterismo cardíaco direito como ferramenta diagnóstica para hipertensão pulmonar na ICFEP

A avaliação abrangente da hemodinâmica cardiovascular pelo CCD é importante não apenas porque pode definir o diagnóstico de ICFEP quando os métodos não invasivos foram inconclusivos, mas também por sua capacidade de adicionar informações críticas que podem modificar a compreensão clínica, a abordagem terapêutica e determinar prognóstico.

Um aspecto que merece destaque é a Hipertensão Pulmonar (HP) na ICFEP. A prevalência de HP na ICFEP diverge entre os estudos, variando de 30% a 80%.^26^ A HP representa um marcador de gravidade da doença e está associada a mau prognóstico.^18,27^

Pacientes com ICFEP, com ou sem HP, apresentam fatores de risco idênticos, comorbidades, características ecocardiográficas do lado esquerdo e pressões de enchimento do lado esquerdo. Além disso, as modalidades não invasivas por si só não conseguem diferenciar a HP pós-capilar da pré-capilar na ICFEP, o que requer CCD.

De fato, a maioria dos pacientes com ICFEP-HP apresenta HP pós-capilar isolada (POAP>15mmHg, PAP média>20mmHg e RVP<2 unidades Wood).^27^ Entretanto, à medida que a doença progride, a congestão crônica levará a outras alterações funcionais e estruturais no sistema vascular pulmonar,^28^ resultando na combinação de HP pós e pré-capilar, definida como POAP>15mmHg, PAP média>20mmHg e RVP>2 Unidades de madeira. Esse aumento adicional na pressão arterial pulmonar levará à disfunção do ventrículo direito e a anormalidades nas trocas gasosas que afetam a sobrevida global desses pacientes.^29,30^

### Cateterismo cardíaco direito para diagnóstico de fenótipo modificado por exercício HFpEF

**Figura 4:**
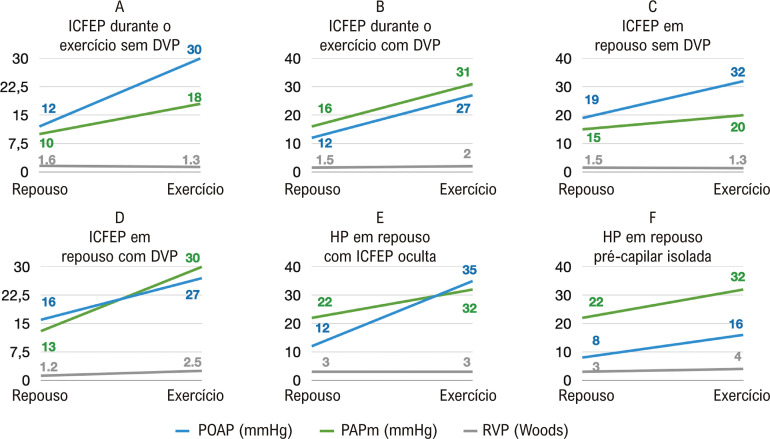
– Dados ilustrativos para representar os perfis hemodinâmicos de exercício da ICFEP(A-E) e Hipertensão Pulmonar (HP). POAP: Pressão de oclusão da artéria pulmonar; PAPm: pressão da artéria pulmonar média; HP: hipertensão pulmonar; DVP: doença vascular pulmonar; RVP: resistência vascular pulmonar.

O desafio do exercício também pode fornecer mais informações sobre a fenotipagem da ICFEP (Figuras 4A-4D).^13,28^ Pacientes com ICFEP sem evidência de HP em repouso podem apresentar uma resposta anormal na RVP durante o exercício (Figura 4A). Isto é indicativo de doença vascular pulmonar latente na ICFEP.^28,31^ Dados recentes mostram que a doença vascular pulmonar latente tem implicações terapêuticas, uma vez que esses pacientes tiveram uma pior resposta ao dispositivo de shunt atrial.^31^

Por outro lado, pacientes com perfil hemodinâmico de repouso de HP pré-capilar (POAP<15mmHg, PAP média>20mmHg e RVP>2 unidades Wood) podem apresentar um aumento desproporcional da POAP durante o exercício quando comparado à pressão atrial direita, atingindo valores de POAP ≥ 25mmHg.^8^ Esse fenótipo combina Doença Vascular Pulmonar (HP pré-capilar) e ICFEP oculta (Figura 4E).^28^

O reconhecimento destes padrões melhorará a nossa compreensão dos mecanismos da intolerância ao exercício nestes pacientes. Além disso, pode trazer implicações terapêuticas significativas.

Laboratórios de hemodinâmica de todo o mundo enfatizam cada vez mais a importância do CCD com exercício, o que representa um passo importante para melhorar a nossa compreensão da ICFEP e formular estratégias individualizadas que pode impactar os desfechos destes pacientes.
