# Supplementary material for: Avançando no Diagnóstico e Tratamento da Insuficiência Cardíaca com Fração de Ejeção Preservada: Um Alerta sobre a Necessidade do Estudo Hemodinâmico com Exercício
Source: Arq Bras Cardiol. 2024 Jul 31;121(7):e20230845. [Article in Portuguese] doi: 10.36660/abc.20230845 (PMC11464094; doi:10.36660/abc.20230845)
Supplement: Translation

# Translation: Advancing the Diagnosis and Management of Heart Failure with Preserved Ejection Fraction: A Call for Exercise Hemodynamics

**DOI:** 10.36660/abc.20230845i

**Authors:** Renato de Aguiar Hortegal, Fausto Feres

**Affiliations:** 1 Brazil Instituto Dante Pazzanese de Cardiologia, São Paulo, SP – Brazil

**Keywords:** Heart Failure, Heart Function Tests, Echocardiography

## Introduction

Heart failure (HF) is a progressive medical condition that affects approximately 1-3% of adults, with a significant increase in prevalence in older age groups.^1^ More than half of HF patients have a left ventricular ejection fraction (LVEF) of ≥ 50%, known as heart failure with preserved ejection fraction (HFpEF). While the overall number of HF cases seems to be stable or even declining, the incidence of HFpEF continues to rise.^2^ HFpEF patients differ significantly from those with reduced ejection fraction regarding pathophysiology, diagnostic evaluation, and treatment. The idiosyncratic pathophysiological heterogeneity and multiorgan dysfunction in addition to diverse clinical presentation will require tailored approaches for this population.

Taking these aspects into account, a specialized unit catering to the HFpEF population was founded in October 2020 at the Instituto Dante Pazzanese de Cardiologia, a tertiary and quaternary public healthcare center located in São Paulo, Brazil. To date, this facility has successfully conducted over 5000 outpatient visits, predominantly for patients presenting dyspnea on exertion.

Within this context, we have encountered a significant challenge currently faced in the Cardiology field:^3^ devising methodologies for conducting effective and practical clinical assessments to establish the diagnosis of HFpEF.

The current diagnostic criteria exhibit considerable heterogeneity and lack of interchangeability.^4,5^ Indeed, the lack of an accurate diagnosis may have hindered the assessment of several therapeutic interventions already tested in multicentric studies. These aspects from the research field have direct implications for how physicians can manage the complaints of shortness of breath encountered in their daily practice among patients with suspected HFpEF.

### The Use of HFpEF Scores

Patients with HFpEF may diverge from the classical HF presentation, as approximately 50% of them exhibit a phenotype characterized by exercise-induced left atrial hypertension, manifesting symptoms exclusively during exertion, without current or previous signs of fluid overload upon clinical examination or prior hospitalization.^6,7^

To further complicate matters, 20-35% of HFpEF patients have normal levels of natriuretic peptides,^1,8^ 29% have no structural abnormality in echocardiography,^4^ and the presence of diastolic dysfunction on echocardiography is neither specific nor sufficient to make a conclusive diagnosis.^1,6^

In this context, two recently developed scoring systems have been introduced with the aim to diagnose HFpEF.^9,10^ The H_2_FPEF score is a nomogram validated against invasive hemodynamics that estimates the probability of HFpEF through the assessment of clinical and Doppler features, with an assumed a pretest probability of 64% (Figure 1). On the other hand, HFA-PEFF is a score system (Figure 2) proposed with a *post-hoc validation* in two large cohorts.^11^

**Figure 1:** – H2FPEF score to estimate the probability of HFpEF.

**Figure 2:** – HFA-PEFF score to evaluate the diagnosis of HFpEF. HFA-PEFF: Heart Failure Association-PEFF; NP: natriuretic peptide; e’: early diastolic mitral annular velocity; E: early transmitral flow velocity; TRV: tricuspid regurgitation velocity; GLS: left ventricular global longitudinal strain; LAVI: left atrial volume index; LVMI: left ventricular mass index; RWT: relative wall thickness; LV: left ventricle; SR: sinus rhythm; NT-proBNP: N-terminal pro-B-type natriuretic peptide; BNP: B-type natriuretic peptide; AF: atrial fibrillation. * Values should adjust to e’<5cm/s and e’ lateral<7cm/s if patients has age>75years/old.

The incorporation of these scoring systems into official cardiology documents holds significant importance, as it contributes to the standardization and traceability of diagnostic evaluations. However, it is important to note that there is limited data regarding the external validation and agreement of these scores.

Preliminary data from our unit, derived from a convenience sample of 320 subjects with dyspnea NYHA grade ≥2 and suspected HFpEF, yielded valuable insights. Most of the patients evaluated displayed scores denoting an intermediate probability of HFpEF. Notably, the H_2_FPEF score exhibited a significantly higher proportion of intermediate probability compared to the HFA-PEFF score (69% vs. 49%, respectively) (Figure 3).

**Figure 3:** – Distribution of HFpEF probability among patients according to HFA-PEFF and H*2*FPEF scores.

Other authors have also reported other limitations of these scores: *Churchill* et al.^12^ reported a cohort of 156 subjects with chronic dyspnea and LVEF≥ 50% submitted to an invasive cardiopulmonary test (iCPET), where it was found an HFA-PEFF score with low probability in 28%, intermediate in 58%, and high in 14%; The H_2_FPEF was low in 32%, intermediate in 61% and high in 7% (Figure 3). Furthermore, the authors reported a false negative rate of 25% and 28% for low probability in HFA-PEFF and H_2_FEPEF, respectively.

These findings have two important implications: 1) further studies are needed to evaluate how to improve the overall performance of the scores, especially for individuals with low and intermediate scores and 2) most patients with suspected HFpEF will likely require exercise tests or alternative hemodynamic stress methods, such as preload and afterload challenges.^13,14^ Recent guidelines recommend Diastolic Stress Test Echocardiography and/or Exercise Right Heart Catheterization in these cases.^1,10^

### Limitations of non-invasive methods to diagnose HFpEF

Diastolic Stress Test with Echocardiography is the first-choice method to study suspected HFpEF during exercise non-invasively.^15,16^

However, this method has significant limitations: the main echocardiographic variable, the E/e’ ratio, may have measurement issues during peak exercise by up to 20% of cases. Furthermore, it is reported that the E/e’ ratio measured during exercise can generate false-positive results in up to 29% of cases, in addition to having suboptimal accuracy.^17^

Although the European Guideline^10^ recommends recalculating scores by adding 2 points (when E/e’ exercise >14 + exercise tricuspid velocity < 3.4 m/s) or 3 points (when E/e’ during exercise >14 +exercise tricuspid velocity > 3.4 m/s) to the calculated HFA-PEFF score, this approach lacks evidence-based support.

While these limitations can hinder the use of Diastolic Stress Echocardiography in defining HFpEF diagnosis, it remains a significant component within the broader multimodality approach for patients with suspected HFpEF.

Gas exchange analysis through cardiopulmonary exercise testing (CPET) stands as the gold standard for non-invasively evaluating functional capacity. This method can explore the interplay between lung mechanics and cardiopulmonary interactions in the context of muscle weakness.^18^ Moreover, recent data suggests that the abnormalities in oxygen kinetics may have a close relationship with impairments in myocardial mechanics.^15,19^

Although non-invasive CPET may have limitations in distinguishing HFpEF from non-cardiac dyspnea in some cases, it plays a valuable role as a diagnostic tool.^18^

### Right heart catheterization as a diagnostic tool for HFpEF

Given the limitations of non-invasive diagnostic modalities, a pivotal factor that may provide critical information is the unique hemodynamic definition of HFpEF syndrome. This definition states HFpEF as the inability of the cardiovascular system to maintain adequate cardiac output at normal filling pressures at rest or during exercise.

Right Heart Catheterization (RHC) is the gold standard for diagnosing HFpEF, due to its ability to measure pulmonary artery wedge pressure (PAWP).^20-22^ The procedure carries a low risk of complications, with reported rates of less than 1%. Additionally, it has demonstrated excellent diagnostic performance.^22^

Under resting conditions, a PAWP ≥ 15mmHg obtained through the Swan Ganz catheter indicates left atrial hypertension. The PAWP, ultimately, reflects the retrograde transmission of mean pressure from the left atrium to the pulmonary capillary.^23^ When elevated, even transiently, this can lead to pulmonary congestion.

However, the normality of PAWP measured at rest does not rule out the diagnosis of HFpEF. Indeed, many patients will need exercise stress to identify hemodynamic abnormalities. After rest measurements, the patient and starts the exercise test with the cycle ergometer, then measurements of PAWP should be taken at intervals of 2-3 minutes. If PAWP reaches a value ≥ 25mmHg, the diagnosis of HFpEF is defined.^22^

An alternative definition considers assessing the rise in PAWP during exercise (slope PAWP) in relation to the increase in cardiac output (slope CO). A ratio slope of PAWP/slope of CO > 2 is indicative of HFpEF.^24^

It is important to mention that exercise RHC requires a complex set-up and demands specialized expertise for data acquisition and interpretation. Moreover, notable variations exist in the protocols (upright vs. supine) and standardization methods for measuring PAWP, such as the reference point (mid-A wave end of expiration vs. mean during the respiratory cycle).^22^

If constraints prevent the execution of exercise RHC, alternative stress during RHC, such as a preload challenge (fluid challenge), may be valuable. A PAWP ≥ 18mmHg induced by passive leg raising or intravenous administration of saline at 7 mL/Kg is diagnostic of HFpEF.^20,22^

### Using right heart catheterization to assess impaired peripheral oxygen extraction: A factor contributing to exercise intolerance in HFpEF

In HFpEF, the ability of muscles to extract oxygen from the bloodstream and utilize it for metabolic processes is jeopardized. This impaired peripheral extraction can lead to inadequate oxygen delivery to exercising muscles, resulting in early fatigue, reduced exercise capacity, and dyspnea.^25^

Assessing oxygen saturation in the pulmonary artery (Sa_o2_) and mixed venous oxygen saturation (Sv_o2_) provides information about oxygen extraction in HFpEF patients. Reduced Sa_o2_ and increased Sv_o2_, indicating impaired utilization of oxygen during exercise. These measurements can help identify patients with HFpEF who exhibit impaired oxygen extraction despite preserved systemic oxygen levels.

Integrating the hemodynamic parameters obtained from RHC makes it possible to assess various physiological parameters during exercise. These parameters include cardiac output, stroke volume, systemic and pulmonary vascular resistance (PVR), pulmonary arterial pressure (PAP), and peripheral oxygen extraction. The Fick equation can be used to evaluate each component that may impact exercise capacity:^25^


$$0.29(0.80)\times 1,000/{mm}^{3}$$

Where VO_2_ represents oxygen consumption, CO represents cardiac output, Cao_2_ represents arterial oxygen content, Cvo_2_ represents venous oxygen content, SV represents stroke volume, HR represents heart rate, Hb represents hemoglobin, Sao_2_ represents arterial oxygen saturation, and Svo_2_ represents venous oxygen saturation.

### Right heart catheterization as a diagnostic tool for pulmonary hypertension in HFpEF

The comprehensive evaluation of cardiovascular hemodynamics by RHC is important not only because it can define the diagnosis of HFpEF when the non-invasive methods were inconclusive but rather it can add critical information that can modify the clinical understanding, evaluation of the therapeutic approach and determine the prognosis.

One aspect that deserves focus is Pulmonary Hypertension (PH) in HFpEF. The PH prevalence in HFpEF diverges among the studies, ranging from 30% to 80%.^26^ PH represents a marker of disease severity, and it is associated with poor prognosis.^18,27^

Patients with HFpEF, both with or without PH, exhibit identical risk factors, comorbidities, left-sided echocardiographic features, and left-side filling pressures. Moreover, non-invasive modalities alone cannot differentiate post-capillary from pre-capillary PH in HFpEF, which requires RHC.

Indeed, most HFpEF-PH patients display isolated post-capillary PH (resting PAWP>15mmHg, mean PAP>20mmHg, and PVR<2 Wood units).^27^ However, as the disease progresses, chronic congestion will lead to other functional and structural changes in the pulmonary vascular system,^28^ resulting in the combined post, and pre-capillary PH, defined as PAWP>15mmHg, mean PAP>20mmHg, and PVR>2 Wood units. This additional increase in pulmonary arterial pressure will lead to right ventricle dysfunction and gas exchange abnormalities that affect the overall survival of those patients.^29,30^

### Right heart catheterization for diagnosing exercise-modified phenotype HFpEF

The exercise challenge can also provide further information on phenotyping HFpEF (Figuras 4A-4D).^13,28^ Patients without evidence of PH at rest may exhibit abnormal responses in pulmonary circulation during exertion, such as paradoxical elevation of PVR during exercise (Figure 4A). This is indicative of latent pulmonary vascular disease in HFpEF.^28,31^ Recent data showed that latent pulmonary vascular disease has therapeutic implications as those patients responded worse to an atrial shunt device.^31^

**Figure 4:** – Illustrative data to represent the exercise hemodynamic profiles of HFpEF (A-E) and Pulmonary Hypertension (PH). PAWP: pulmonary artery wedge pressure; PAPm: mean pulmonary artery pressure; PH: pulmonary hypertension; PVD: pulmonary vascular disease; PVR: pulmonary vascular resistance.

Conversely, patients with a resting hemodynamic profile of pre-capillary PH (resting PAWP<15mmHg, mean PAP>20mmHg, and PVR>2 Wood units) can display a disproportionate increase in PAWP during exercise when compared to right atrial pressure reaching values of PAWP≥25mmHg.^8^ This phenotype combines Pulmonary Vascular Disease (pre-capillary PH) and occult HFpEF (Figure 4E).^28^

Recognizing these patterns will enhance our understanding of the mechanisms behind exercise intolerance in these patients. Besides, it may carry significant therapeutic implications.

Several leading healthcare centers worldwide are placing greater emphasis on exercise RHC. This emphasis represents a crucial step in enhancing the understanding of HFpEF and in formulating tailored strategies aimed at improving quality of life and clinical outcomes.
